# Effect of pemafibrate (K-877), a novel selective peroxisome proliferator-activated receptor α modular (SPPARMα), in atherosclerosis model using low density lipoprotein receptor knock-out swine with balloon injury

**DOI:** 10.1371/journal.pone.0241195

**Published:** 2020-11-17

**Authors:** Hirokazu Konishi, Katsumi Miyauchi, Akira Onishi, Shunichi Suzuki, Daiichiro Fuchimoto, Jun Shitara, Hirohisa Endo, Hideki Wada, Shinichiro Doi, Ryo Naito, Manabu Ogita, Tomotaka Dohi, Takatoshi Kasai, Hiroyuki Daida

**Affiliations:** 1 Department of Cardiovascular Medicine, Juntendo University Graduate School of Medicine, Tokyo, Japan; 2 Laboratory of Animal Reproduction, Department of Animal Science and Resources, College of Bioresource Sciences, Nihon University, Kanagawa, Japan; 3 Division of Animal Sciences, Institute of Agrobiological Sciences, National Agriculture and Food Research Organization (NARO), Ibaraki, Japan; The Pennsylvania State University, UNITED STATES

## Abstract

**Background:**

Peroxisome proliferator-activated receptor α (PPARα) is a nuclear receptor that has key roles of lipid metabolism and inflammation. The PPARα may affects the initiation and progression of atherosclerosis by reducing inflammatory responses. Pemafibrate (K-877) is a novel selective PPARα modulator (SPPARMα), which was designed to possess higher PPARα potency and selectivity than existing PPARα agonists. The aim of this study is to evaluate the effect of pemafibrate on vascular response in coronary atherosclerosis model using low density lipoprotein receptor knock-out (LDLR-KO) pigs with balloon injury.

**Methods and results:**

Ten LDLR-KO pigs were randomly allocated to two groups [pemafibrate (n = 5) and control (n = 5)] and fed with a diet containing 2.0% cholesterol and 20% lard throughout the study. Balloon injury was created in 40 coronary segments two weeks after starting the oral administration of pemafibrate or placebo. Necropsy was conducted 8 weeks later. Coronary artery sections were reviewed to evaluate lesion progression and the mRNA expression levels for C-Jun, NFκ B, CCL2, CCR7, CD163 and MMP9 determined using real-time RT-PCR. LDL cholesterol at baseline was about 700 mg/dL. The mean ratio of macrophages to plaque area was significantly lower in pemafibrate group compared with control one (7.63±1.16 vs 14.04±4.51, P = 0.02) whereas no differences were observed in intimal area between groups. The mRNA levels of C-Jun, NFκB and MMP9 were significantly decreased in pemafibrate group.

**Conclusions:**

Pemafibrate was associated with inhibition of inflammatory responses in coronary artery atherosclerosis model using LDLR-KO swine with balloon injury.

## Introduction

Peroxisome proliferator-activated receptor α (PPARα) is a nuclear receptor that has key roles of lipid metabolism and inflammation [1]. The PPARα may affect the initiation and progression of atherosclerosis by reducing inflammatory responses [2]. Fibrates are currently used to control dyslipidemia, which act as PPARα ligand that regulate the expression of numerous genes important in lipid metabolism [3]. Since the use of fibrates reduced cardiovascular events in patients with elevated triglyceride and low high-density lipoprotein cholesterol (HDL-C) in several trials, fibrates are recommended to manage these patients [4]. However, there are some limitations of the use of fibrates that leads an elevation of creatine kinase, alanine aminotransferase and serum creatinine levels in monotherapy and increased in combination with statins [5].

Pemafibrate (K-877) is a novel selective PPARα modulator (SPPARMα), which was designed to possess higher PPARα potency and selectivity than existing PPARα agonists (such as fibrates) [6,7]. In a clinical data, pemafibrate improved triglyceride, HDL-C and other lipid parameters compared with fenofibrate in patients with dyslipidemia [8]. In a preclinical data, pemafibrate reduced markers of inflammation and macrophages in the aortic crosses in apolipoprotein E2 knock-In mice [9]. However, there are few studies regarding the effect of pemafibrate on the vascular response, especially in coronary artery.

The aim of this study is to evaluate the effect of pemafibrate in atherosclerosis model using low density lipoprotein receptor knock-out (LDLR-KO) pigs with balloon injury.

## Methods

### Ethics statement

Experiment using gene recombinant animals were performed in agreement with the Gene Recombination Experiment Security Committees University of Juntendo University (No. DNA22-44) and the National Institute of Agrobiological Science (No. 500035). Animal Care an Use Committee of Juntendo University (No. 1036) and the National Institute of Agrobiological Science (No. H18-038) approved this study and the experiment were conducted in accordance with the NIH guidelines (Guide for the care and use of laboratory animals). All pigs were housed and monitored with veterinary care at the Center for Biomedical Research Resources, Juntendo University.

### Animals

**Fig 1:**
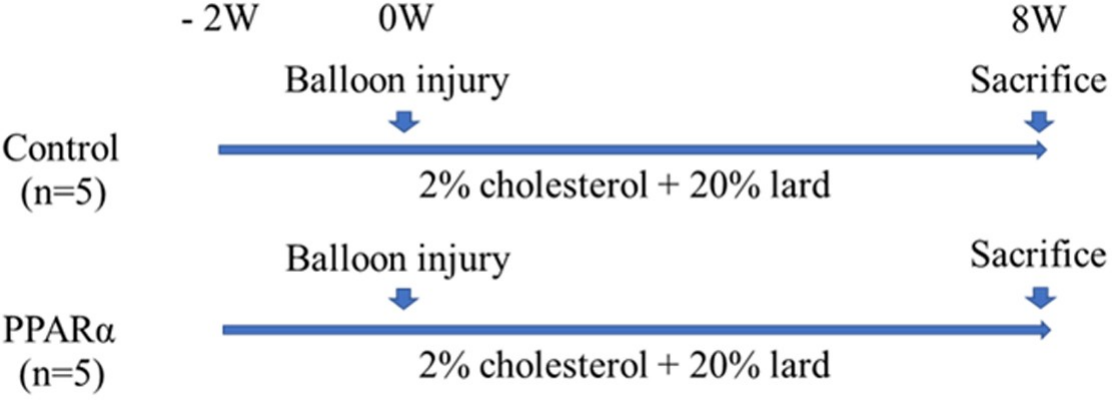
Study protocol. Ten two-month-old LDLR-KO pigs were randomly allocated to two groups and fed with a diet containing 2.0% cholesterol + 20% lard throughout the study. The coronary arteries were injured using balloons and then the pigs were sacrificed at 8 weeks later.

Cloned LDL-R-targeted pigs were produced as previously described [10–12]. Ten juvenile LDLR-KO pigs (Sus scrofa, aged 2 to 3 months and weighing 20–30 kg) were randomly allocated into control groups (n = 5) and pemafibrate groups (n = 5). Pemafibrate provided by Kowa Company was orally administered once daily at doses of 1mg/kg. All pigs were fed with a diet comprising of 2.0% cholesterol plus 20% lard throughout the study to maintain hypercholesterolemia. Angioplasty was performed in the left anterior descending (LAD) and left circumflex coronary artery (LCX) of both groups to accelerate coronary plaque development (Fig 1). All pigs euthanized at 8 weeks after balloon injury. Blood samples were obtained at baseline and 8 weeks after balloon injury.

### Procedures

The pigs were anesthetized using ketamine (30mg/kg) and xylazine (3mg/kg) intramuscularly and maintained with isoflurane by ventilator after intubation. Cut-down was performed on carotid artery and 6Fr arterial sheath inserted as for vascular access. All pigs received aspirin (330mg) two days before procedure and continued. After coronary angiography (CAG) with systemic heparinization (5000 IU/body), LAD and LCX were dilated three times for 20 seconds using an oversize balloon (Angioscurpt, Volcano Japan) at a 1.1–1.2 ratio of balloon to artery diameter.

### Tissue harvesting

8 weeks after angioplasty, the pigs were euthanized using intravenous barbiturate commercial euthanasia solution by ear vein (50 mg/kg). The LAD and LCX were harvested and perfused with 10% buffered formalin. 4 sections injured by balloon (LAD proximal, LAD distal, LCX proximal and LCX distal) of coronary artery per pig were paraffin embedded and stained with hematoxylin-eosin, Elastic van Gieson and Azan. The sections were examined by microscopy and their areas measured using a KS-400 image-analysis system (Carl Zeiss vision GmbH, Hallbermoss, Germany). Injured areas encroached to the external elastic lamina (EEL), the internal elastic lamina (IEL) and lumen areas were measured using computer-assisted digital planimetry. Intimal (plaque) areas was calculated using computer-assisted digital planimetry as the IEL area minus lumen area.

### Immunohistochemistry

Paraffin-embedded slides were deparaffinized, rehydrated and then antigen retrieved by heat induction. A goat polyclonal cathepsin-S antibody (Santa Cruz Biotechnology Inc.), a rabbit polyclonal MCP1 antibody (Bioss Inc.) and a rabbit polyclonal MMP9 antibody (Bioss Inc.) were applied. Tissue sections were visualized using NeXES IHC automatic immunostainer (Ventana Medical Systems, Tucson, AZ, USA) and a standard 3’, 3’-diaminobenzidine (DAB) detection kit (Ventana Medical Systems) and counterstained with hematoxylin. The sections were dehydrated, placed in xylene and coverslipped. The ratio (%) of the cathepsin S, MCP 1 and MMP 9 positive area was determined under high-power magnification (x 400) using a KS-400 image analyzing system (Carl Zeiss Vision GmbH, Hallbermoss, Germany).

### Real time reverse transcription—Polymerase chain reaction

RNA extraction was performed using the RNeasy Plus Universal Kits according to the manufacturer’s instructions. Reverse transcription into cDNA was performed using the High Capacity cDNA Reverse Transcription Kits according to manufacturer’s instructions. The cDNA was amplified on a 7500 Fast Realtime PCR System (Thermo Fisher Scientific) using swinespecific Taqman probe sets (all purchased from Thermo Fisher Scientific) for C-Jun (Assay ID Ss03382061_u1), NFκB (Assay ID Ss03388575_m1), CCL2 (Assay ID Ss03394377_m1), CCR7 (Ss03377879_s1), CD163 (Assay ID Ss03393162_u1) and MMP9 (Assay ID Ss03392100_m1) according to the manufacturer’s protocol under the following conditions: 95°C (20 seconds) for 1 cycle followed by 40 cycles of: 95°C (3 seconds) and 60°C (30 seconds). All samples were run in triplicate. Target gene expression was related to the expression levels of RPL27 mRNA in each specific sample (Assay ID Ss03385714_g1).

### Statistical analysis

All data are presented as the mean ± S.D. Groups were compared using student’s unpaired two-tailed t-test. Categorical variables were percentages and compared by X^2^ test. P- value < 0.05 was considered to indicate statistical significance. All data were statistically analyzed using JMP 8.0 (SAS Institute Inc., Cary, NC, USA).

## Results

### Serum lipids

Mean baseline levels of LDL-C (control group and pemafibrate group) were 722.5±172.7 mg/dL and 678.2±165.3 mg/dL, respectively, which was similar level between two groups (P = 0.64) (Table 1). The serum levels of LDL-C maintained high level (700–800 mg/dL) during the study period in both groups. The serum levels of triglyceride and HDL-C were similar between two groups.

**Table 1:** Baseline and time of sacrifice data of body weight and lipid profiles.

	Control (n = 5)	PPARα (n = 5)	P value
< Body weight (kg) >			
At balloon injury	43.8 ± 7.5	39.8 ± 8.3	0.48
8 weeks	63.3 ± 10.2	58.7 ± 11.7	0.55
< Lipid profiles (mg/dl) >			
At balloon injury			
TC	763.2 ± 177.1	718.4 ± 160.4	0.64
LDL-C	722.5 ± 172.7	678.2 ± 165.3	0.67
HDL-C	36.5 ± 9.5	29.4 ± 5.8	0.42
TG	59.8 ± 24.1	63.4 ± 17.1	0.79
8 weeks			
TC	814.2 ± 90.8	789.2 ± 71.1	0.66
LDL-C	766.2 ± 84.4	753.4 ± 76.3	0.81
HDL-C	37.7 ± 5.6	32.8 ± 5.5	0.46
TG	50.7 ± 20.2	47.6 ± 12.9	0.78

TC, total cholesterol; LDL-C, low density lipoprotein cholesterol; HDL-C, high density lipoprotein cholesterol; TG, triglyceride.

### Coronary artery lesions 8 weeks after balloon injury

**Fig 2:**
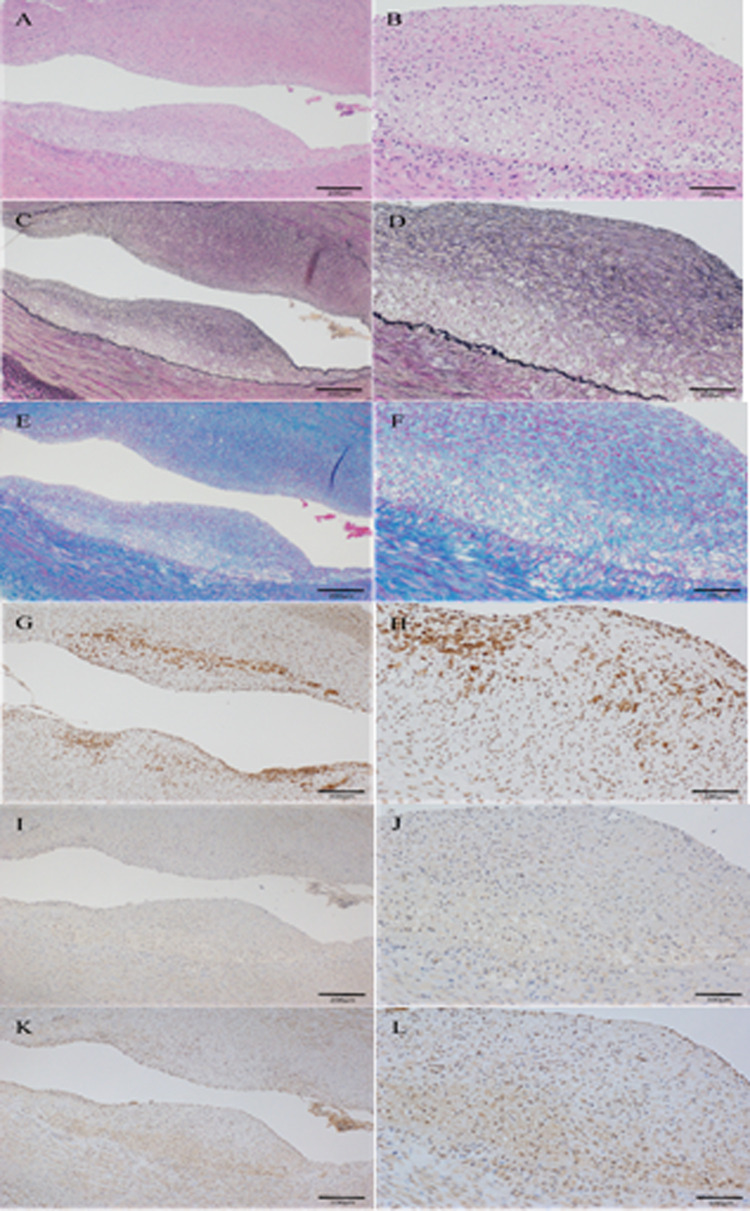
(A-L): Representative coronary artery section of LDLR-KO pig at 8 weeks after balloon injury in control groups. A and B, hematoxylin-eosin; C and D, Elastica van Gieson; E and F, Azan; G and H, cathepsin S immunohistochemical staining; I and J, MCP 1 immunohistochemical staining; K and L, MMP 9 immunohistochemical staining. Scale bar, 200μm on the left panel and 100μm on the right panel.

**Fig 3:**
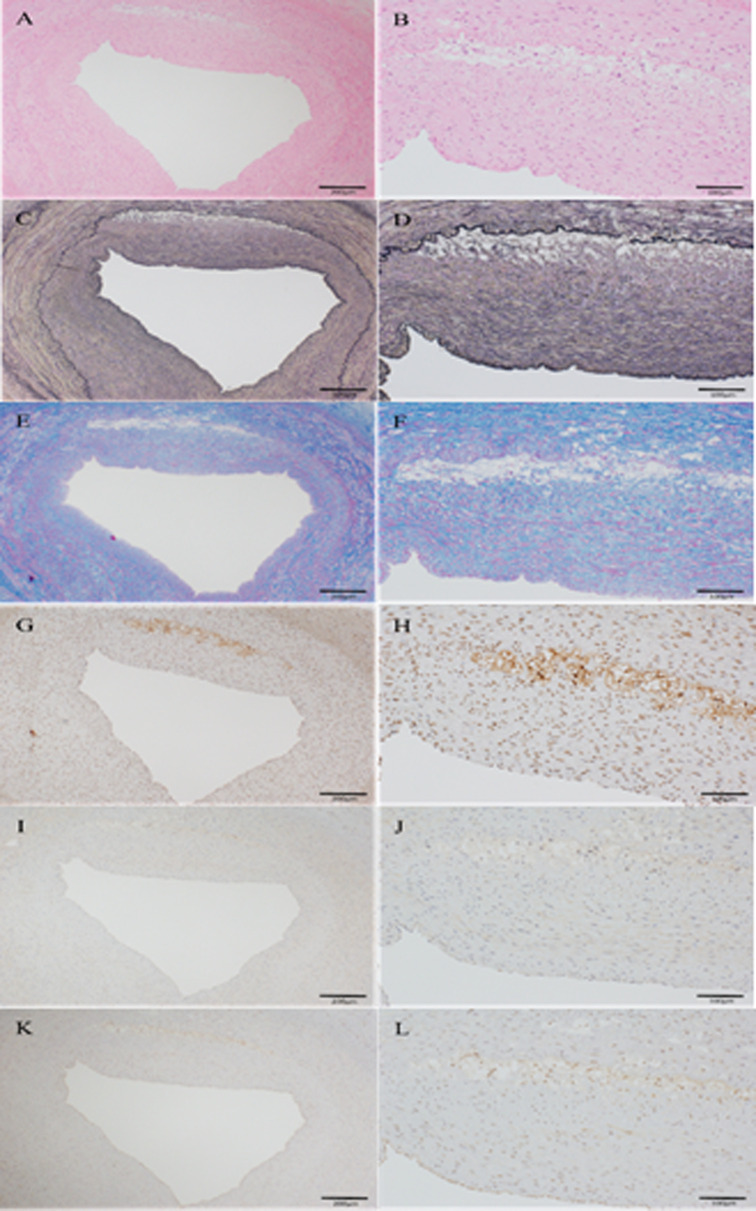
(A-L): Representative coronary artery section of LDLR-KO pig at 8 weeks after balloon injury in PPARα groups. A and B, hematoxylin-eosin; C and D, Elastica van Gieson; E and F, Azan; G and H, cathepsin S immunohistochemical staining; I and J, MCP 1 immunohistochemical staining; K and L, MMP 9 immunohistochemical staining. Scale bar, 200μm on the left panel and 100μm on the right panel.

**Fig 4:**
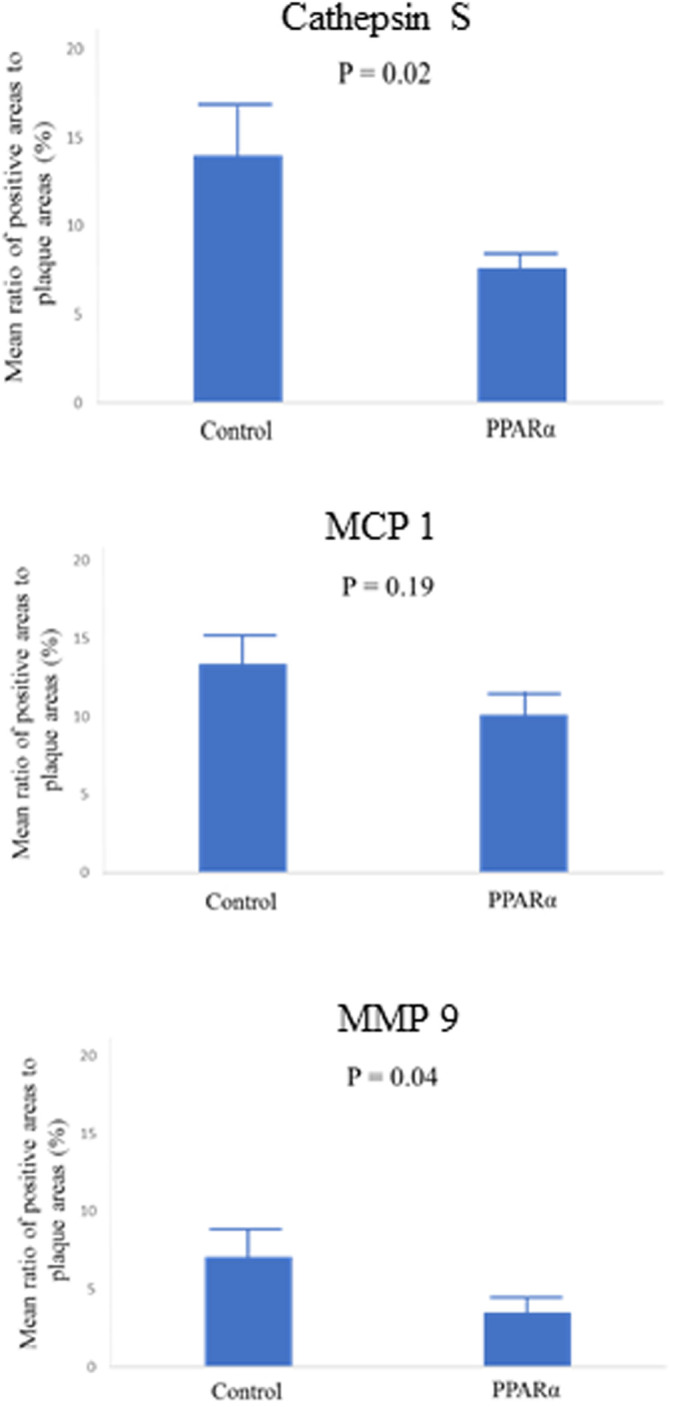
Ratio of areas positively stained for cathepsin S, MCP 1 and MMP 9.

Table 2 showed pathological findings. 40 coronary artery sections [4sections (LAD proximal, LAD distal, LCX proximal and LCX distal) per a pig] were obtained 8 weeks after balloon injury to evaluate lesion progression. The mean intima to media ratio was no different between two groups (P = 0.71). However, Pemafibrate attenuated cathepsin S, MCP1 and MMP9 production. The mean ratio of macrophages (cathepsin S) to plaque area was significantly lower in pemafibrate group compared with control one (7.63±1.16 vs 14.04±4.51, P = 0.02). The mean ratio of MMP9 to plaque area was also significant differences in permafibrate group compared with control one (3.51±1.41 vs 7.09±2.98, P = 0.04) (Figs 2–4).

**Table 2:** Pathological values at 8 weeks after balloon-induced arterial injury.

	Control (n = 20)	PPARα (n = 20)	P value
Vessel area (mm2)	4.06 ± 0.42	3.67 ± 0.62	0.32
Intima area (mm^2^)	1.70 ± 0.51	1.46 ± 0.43	0.46
Medial area (mm2)	2.18 ± 0.46	1.94 ± 0.34	0.38
Intima / Media ratio	0.77 ± 0.10	0.74 ± 0.09	0.71

### The mRNA levels of coronary artery

**Fig 5:**
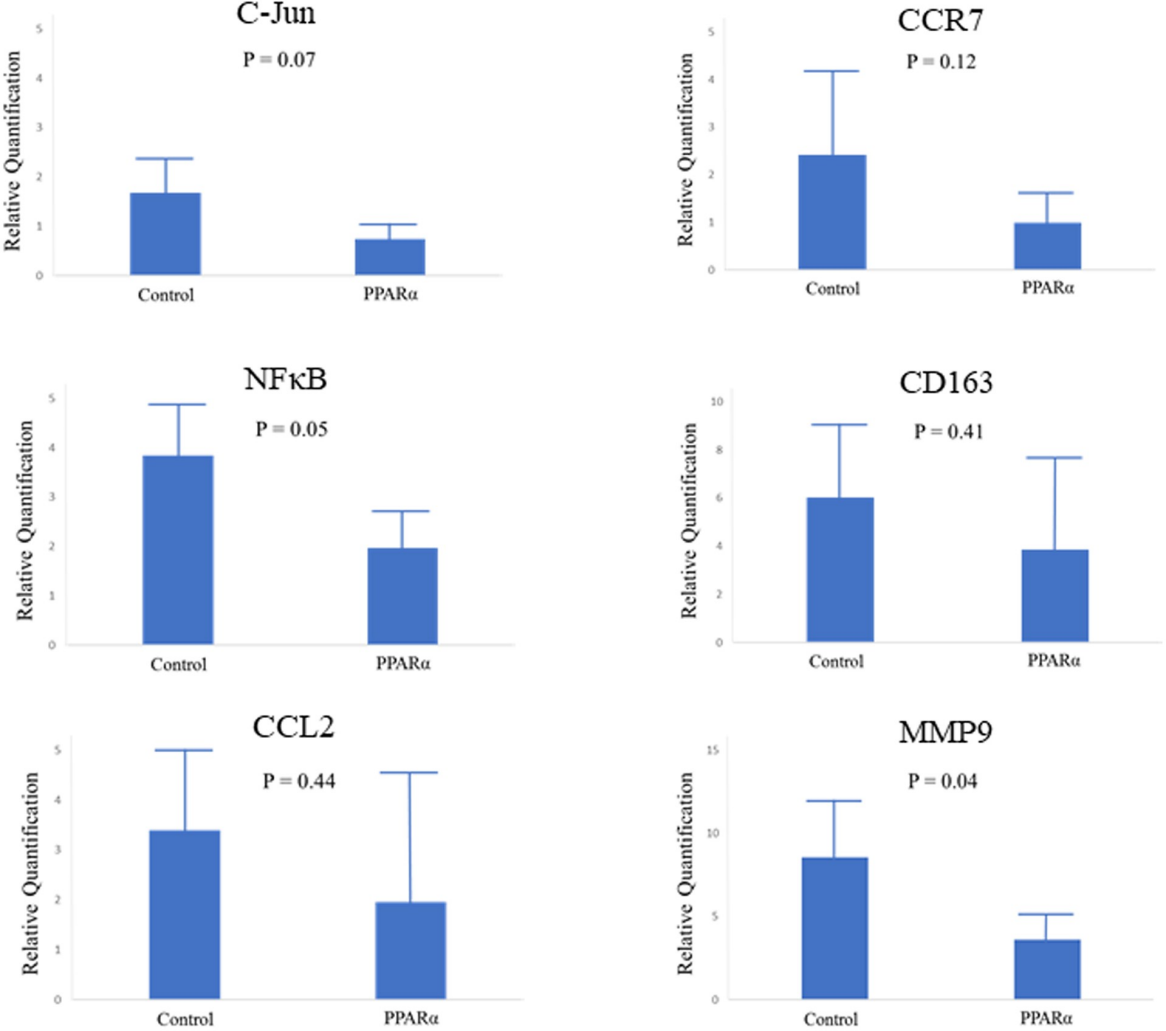
Gene expression levels of C-Jun, NFκB, CCL2, CCR7, CD163 and MMP9.

Fig 5 showed all inflammatory parameters were attenuated in pemafibrate group. The mRNA levels of C-Jun (AP-1), NFκB and MMP9 were significantly decreased in pemafibrate group (P = 0.07, 0.05 and 0.04). CCR7 (M1 macrophage) trends to be attenuated in permafibrate group, whereas CD163 (M2 macrophage) wasn’t changed.

## Discussions

Pemafibrate (K-877) is a novel selective PPARα modulator (SPPARMα), which was designed to possess higher PPARα potency and selectivity than existing PPARα agonists (such as fibrates) [6,7]. In the present study, the effect of pemafibrate on vascular response was examined in atherosclerosis model using LDLR-KO pigs with balloon injury. Swine coronary arteries are anatomically and physiologically similar to humans [13]. We previously established a model of accelerated coronary atherosclerosis using LDLR-KO pigs with balloon injury [14]. Since this model might be useful as preclinical tool for evaluating pharmacological action, we use this model in the present study. The finding of this study are as follows: 1) pemafibrate inhibits gene expression of AP-1, NFκB, macrophage and MMP9, 2) pemafibrate attenuated macrophage, MMP9 and MCP1 production in coronary artery. We found pemafibrate was associated with the inhibition of inflammatory responses in coronary artery.

PPARs are a superfamily of nuclear hormone receptors that form complexes with the retinoid X receptor and bind to PPAR response elements on DNA. This process leads to the repression and activation of target genes. During repression, the activated PPAR binds to cytokine-activated transcription factors, such as NFκB and AP-1 [15]. These factors induce the synthesis of proteins which are involved in the inflammatory response. PPARα inhibits this process by preventing transcription and reduce inflammation. PPARα displays anti-inflammatory activities and controls the inflammatory response in the vascular wall [16,17]. Previous preclinical studies have established the pharmacological profile of pemafibrate. Pemafibrate reduced markers of inflammation and macrophages in the aortic crosses as well as aortic atherosclerotic lesion burden in mice [9]. Administration of pemafibrate in mice resulted in the event of vascular disorders, there was reduced neointimal formation and suppressed macrophage accumulation [18]. In the present study, pemafibrate (K-877) interfered the gene expression of NFκB and AP-1, and lead to attenuate macrophage, MMP9 and MCP1 production in coronary artery. The main different and novelty points between the present study and previous studies were using swine and evaluating coronary artery. Since swine coronary arteries are anatomically and physiologically similar to humans, the present study is very useful in clinical practice.

Vulnerable plaques are easy to rupture leading to thrombus formation and vascular occlusion, which cause acute coronary syndrome. These plaques are abundance of macrophages and other inflammatory cells, with scarce of smooth muscle cells in the fibrous cap region [19]. On the other hand, plaques with thick cap are stable and having low clinical events. Maintenance of stable plaques requires a balance between extracellular matrix production and degradation. Matrix metalloproteinase, specifically MMP9, is one of key role for vulnerable plaques. MMP9 causes thin-fibrous cap and plaque rupture [20]. PPARα agonists decrease gene transcription and activity of MMP9 [21]. In the present study, pemafibrate (K-877) interfered the gene expression of MMP9 and lead attenuate MMP9 production in coronary artery. It reveals pemafibrate (K-877) have potential effect to prevent formation of vulnerable plaques.

In current study, pemafibrate (K-877) did not reduce the intimal hyperplasia compared with placebo. In contrast, previous our results showed that fenofibrate inhibited neointimal hyperplasia after angioplasty through inhibition of inflammation in porcine coronary arteries [22]. The differences of the response of intimal hyperplasia between current and previous studies were due to the differences of porcine coronary models though similar pharmacological activation of PPARα. Current study was the use of accelerated coronary atherosclerosis models with LDLR-KO pigs after balloon injury, which consisted of smooth muscle cells (SMCs), macrophages, and inflammation cells and were histologically similar to humans. Previous study was simple balloon injury model, in which intimal hyperplasia consisted of proliferation of SMCs. It was very difficult to inhibit proliferation of such a complicated pathological lesion by single pharmacological intervention, pemafibrate. However, pemafibrate inhibited the inflammatory responses which means that negatively interfering with the inflammatory response at the level of the vascular wall lead to a selective inhibition of the atherogenic process since chronic inflammation is a key role of atherosclerosis.

In the present study, pemafibrate didn’t affect the lipid profile since the serum level of triglyceride was too low in pigs. However, pemafibrate improved triglyceride and HDL-C without side effects compared to placebo and fenofibrate in dyslipidemia patients [8]. Furthermore, these efficacy and safety of pemafibrate in combination with statin were demonstrated in patients with dyslipidemia who had persistently elevated triglyceride as residual cardiovascular disease risk despite statin therapy [23]. Clinical trials have shown the importance of reducing LDL-C levels with statins to prevent adverse events and progressive atherosclerosis. In addition, HDL-C and triglyceride are being focused as residual risk factors for cardiovascular disease in patients who have achieved LDL-C target values. In patients with dyslipidemia, treatment with pemafibrate not only increased HDL-C, apo A-I and apo A-II levels, but also improved HDL function, as shown by increases in prebeta-HDL, smaller HDL particles and macrophage cholesterol efflux capacity [24,25]. The findings of our study and recent clinical data suggest that pemafibrate (K-877) could be unmet medical needs for the treatment of residual cardiovascular disease risk factors.

## Limitations

The present study has limitations. We combined high-cholesterol diet with balloon injury to accelerate the atherosclerotic process and reduce the observation period in a small sample of LDLR-KO pigs. Compared with natural human atherosclerosis lesion, our model produced plaque containing smooth muscle cells driven from balloon vascular injury. It might lead the result the mean intima to media ratio was no different between two groups in the present study. However, the patients with coronary artery disease were treated using balloons and stents (percutaneous coronary intervention), therefore, injury induced model like our study might be helpful to value the effects of pharmacology. The purpose of present study was to evaluate the effect of pemafibrate in atherosclerosis model using low density lipoprotein receptor knock-out (LDLR-KO) pigs. Therefore, we did not compare the difference between pemafibrate and other fibrates in the present study.
